# The Efficacy and Safety of Pelubiprofen in the Treatment of Acute Upper Respiratory Tract Infection: A Multicenter, Randomized, Double-Blind, Non-Inferiority Phase III Clinical Trial Compared to Loxoprofen

**DOI:** 10.3390/jcm14051450

**Published:** 2025-02-21

**Authors:** An Soo Jang, Sang Hoon Kim, Sang Pyo Lee, Moon Jun Na, Kwang Ha Yoo, Chang Han Park, Seong Yeon Park, Byoung Whui Choi

**Affiliations:** 1 Department of Internal Medicine, Soonchunhyang University Bucheon Hospital, Bucheon 14584, Republic of Korea; jas877@schmc.ac.kr; 2 Department of Internal Medicine, Eulji University School of Medicine, Nowon Eulji University, Seoul 01830, Republic of Korea; 3 Department of Internal Medicine, Gachon University Gil Medical Center, Incheon 21565, Republic of Korea; 4 Department of Internal Medicine, Konyang University Hospital, Daejeon 35365, Republic of Korea; 5 Department of Internal Medicine, Konkuk University Medical Center, Seoul 05030, Republic of Korea; 6 Department of Internal Medicine, Sungae Hospital, Seoul 07354, Republic of Korea; 7 Department of Internal Medicine, Dongguk University Ilsan Hospital, Goyang 10326, Republic of Korea; 8 Department of Internal Medicine, Soha Healthcare Center, Chung-Ang University Gwangmyeong Hospital, Gwangmyeong 14353, Republic of Korea

**Keywords:** pelubiprofen, loxoprofen, efficacy, randomized controlled trial, safety, NSAIDs, URTIs, fever

## Abstract

**Background/Objectives:** Acute upper respiratory tract infections (URTIs) are common illnesses that cause significant discomfort due to symptoms such as fever, headache, sore throat, and muscle pain. Non-steroidal anti-inflammatory drugs (NSAIDs) are widely used for symptom relief due to their anti-inflammatory, analgesic, and antipyretic properties. Pelubiprofen, a new NSAID, has not been extensively evaluated for its efficacy and safety in treating URTI-related symptoms, particularly fever. This study aimed to demonstrate that pelubiprofen is not inferior to loxoprofen in reducing fever in patients with URTIs. **Methods**: This phase III, multicenter, randomized, double-blind, parallel-group, active-controlled, non-inferiority trial involved 181 adults with URTI-related fever (≥38.0 °C), who were randomly assigned to receive pelubiprofen or loxoprofen at a 1:1 ratio. The primary end point was decreasing axillary temperature 4 h post-dose. Secondary end points included fever reduction, pain relief based on the visual analog scale (VAS), and safety. **Results:** Of the 181 participants, 130 (pelubiprofen [*n* = 61] and loxoprofen group [*n* = 69]) underwent randomization. The mean reduction in axillary temperature at 4 h post-dose was comparable between the two groups (−0.08 ± 0.62 °C). The lower bound of the 97.5% one-sided confidence interval was −0.30 °C, which is greater than the non-inferiority margin of 0.35 °C, demonstrating the non-inferiority of pelubiprofen to loxoprofen. The secondary outcomes showed no significant differences in efficacy or safety (*p* > 0.05). **Conclusions**: Pelubiprofen is not inferior to loxoprofen in reducing fever associated with URTIs and is a safe and effective treatment option. Registration: (ClinicalTrials.gov identifier: NCT01779271).

## 1. Introduction

Acute upper respiratory tract infections (URTIs) are the most common illnesses, causing symptoms such as fever, headache, sore throat, and muscle aches, leading to significant discomfort and economic burden [1]. Non-steroidal anti-inflammatory drugs (NSAIDs) are widely used to alleviate these symptoms due to their anti-inflammatory, analgesic, and antipyretic effects.

Pelubiprofen, approved by the Korean Ministry of Food and Drug Safety (MFDS) as the 12th new molecular entity in Korea, is a propionic acid-derived NSAID indicated for the relief of symptoms and signs of osteoarthritis in 2007, low back pain in 2010, and rheumatoid arthritis in 2012. Preclinical studies have demonstrated the antipyretic efficacy of pelubiprofen, showing that the inhibitory concentrations for analgesic and antipyretic effects are comparable [2,3]. Considering existing NSAIDs, like ibuprofen and loxoprofen, at doses used for osteoarthritis and URTIs, this study aimed to evaluate the therapeutic efficacy and safety of pelubiprofen at the approved dose (30 mg per dose) for treating fever and pain due to URTIs.

This phase III clinical trial was designed to compare the efficacy and safety of pelubiprofen to those of loxoprofen in alleviating URTI symptoms, focusing on fever reduction, pain relief, and overall safety.

## 2. Materials and Methods

### 2.1. Study Design and Participants

This multicenter, randomized, double-blind, parallel-group, non-inferiority phase III trial was conducted from February 2013 to March 2016 at 10 medical institutions to compare the efficacy and safety of pelubiprofen 30 mg with loxoprofen 60 mg in adults presenting with fever (axillary temperature ≥ 38.0 °C) and symptoms of URTIs. After providing informed consent, eligible participants were randomly assigned to receive either pelubiprofen (30 mg) or loxoprofen (60 mg). Axillary temperature was measured at baseline (pre-dose) and 0.5, 1, 1.5, 2, 3, 4, and 6 h post-dose. Headache, pharyngeal pain/odynophagia, and joint/muscle pain were assessed using a 100 mm visual analog scale (VAS) at 4 and 6 h post-dose. The use of additional antipyretic medication, including salicylates (e.g., Aspirin^®^), pyrines (e.g., Geworin^®^), and acetaminophen (e.g., Tylenol^®^) was not permitted within 6 h post-dose unless clinically justified by the attending physician. The trial was discontinued for participants whose body temperature rose above 40.0 °C after drug administration.

**Figure 1:**
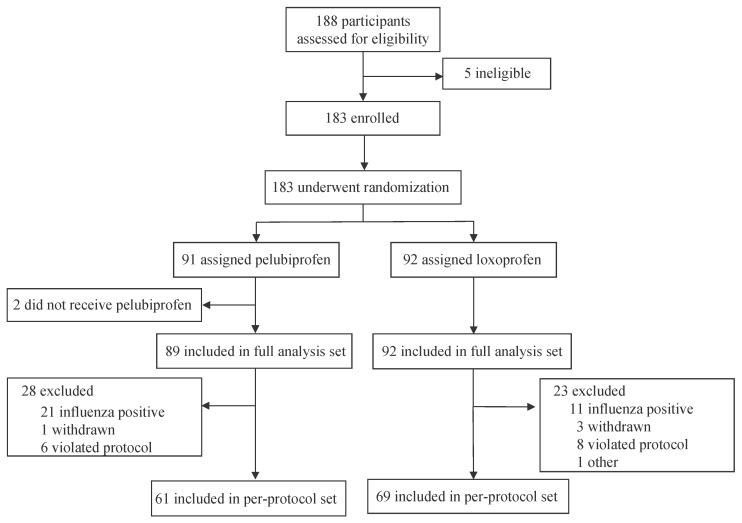
Flow diagram of participant enrollment and allocation.

This study was conducted in full compliance with the guidelines for Good Clinical Practice and the Declaration of Helsinki. The institutional review boards of each medical institution and the MFDS approved the protocol. Written informed consent was obtained from each participant prior to enrollment. No significant amendments were made to the study protocol after the trial commenced. The study was conducted in accordance with the CONSORT 2010 checklist, and the CONSORT flow diagram is shown in Figure 1.

### 2.2. Inclusion and Exclusion Criteria

Participants aged 15 years or older with a clinical diagnosis of URTI and an axillary temperature of 38.0 °C or higher were eligible. Symptom onset had to occur within 48 h before enrollment, and participants were required to abstain from URTI-related medications for at least 8 h prior to the study drug administration. Informed consent was obtained from all participants or their legal guardians.

Exclusion criteria included recent use of antipyretics within 4 h of screening, a history of febrile crisis within the past 6 months, or significant gastrointestinal conditions, such as confirmed peptic ulcer disease requiring ongoing treatment. Other exclusion factors were severe hematologic disorders, hepatic impairment (alanine aminotransferase (ALT) or aspartate aminotransferase (AST) ≥ 2 times the upper limit of normal), renal impairment (serum creatinine ≥ 2 times the upper limit of normal), severe cardiac dysfunction, and uncontrolled hypertension (systolic blood pressure (SBP) ≥ 160 mmHg or diastolic blood pressure (DBP) ≥ 100 mmHg). Individuals with known hypersensitivity to NSAIDs or significant respiratory diseases, including bronchial asthma or chronic obstructive pulmonary disease, and those requiring other medications (e.g., antibiotics) during the trial were also excluded.

### 2.3. Randomization and Blinding

Participants were randomly assigned to either the pelubiprofen or the loxoprofen group using the random permuted block method. Randomization sequences were generated through a validated SAS randomization program (SAS V9.2). The study design incorporated a double-dummy approach to maintain blinding, with randomization codes securely concealed until the trial conclusion. These codes were accessible only under medically necessary circumstances to prevent bias.

### 2.4. Procedures

After obtaining informed consent, participants were randomly assigned to receive either 30 mg of pelubiprofen paired with placebo-matching loxoprofen or 60 mg of loxoprofen with placebo-matching pelubiprofen. Both treatments were administered as single oral doses.

Body temperature was measured using a mercury thermometer of the same model, calibrated, and provided to each institution to ensure standardization. Axillary body temperature was measured in the left axilla, ensuring no functional or anatomical abnormalities and minimal movement during measurement. Temperature was measured by the same examiner whenever possible at the deepest part of the axillary cavity, slightly anterior to the center of the armpit, twice each time. If the difference between these values was ≤0.1 °C, the first value was recorded; if the difference exceeded 0.2 °C, the value was measured again and the value closer to the third value among the first and second measured values was determined to be the temperature.

### 2.5. Outcomes

#### 2.5.1. Efficacy Profiles

The primary outcome was the reduction in axillary temperature at 4 h post-dose. The temperature difference from baseline to 4 h post-dosing was evaluated for each participant.

Secondary outcomes included the area under the curve (AUC) for temperature change over 6 h, the maximum temperature reduction, and the proportion of participants achieving temperature normalization (defined as <37.0 °C). The mean time to temperature normalization was also assessed. Additionally, reductions in VAS scores for headache, pharyngeal pain/odynophagia, and joint/muscle pain at 4 and 6 h post-dose were evaluated.

#### 2.5.2. Safety Profiles

Adverse events (AEs), defined as any undesirable or unintended medical event occurring in subjects who received the study drug regardless of a confirmed causal relationship with the treatment, were documented throughout the study period. Laboratory assessments, including hematology (e.g., white blood count (WBC), red blood count (RBC), hemoglobin, hematocrit, and platelet count), biochemistry (total protein, albumin, bilirubin, AST, ALT, alkaline phosphatase (ALP), creatinine, and glucose), and urinalysis (protein, glucose, RBC, and WBC), were conducted at baseline and 6 h post-dose. Physical examination and vital signs, specifically blood pressure and heart rate, were measured before and after the study drug administration to monitor potential fluctuations.

In addition to monitoring clinical symptoms, any newly emerging symptoms, worsening of pre-existing conditions, or significant changes in laboratory values were classified as AEs and closely monitored and recorded. Participants were observed for 6 h post-dose to promptly identify and manage any immediate adverse effects, ensuring a thorough evaluation of the safety profile of the study drugs.

### 2.6. Statistical Analysis

The primary objective of this clinical trial was to establish the non-inferiority of pelubiprofen compared to loxoprofen in terms of antipyretic effects, specifically the reduction in body temperature post-dose. The required sample size was determined in a previous clinical trial evaluating the antipyretic effects of loxoprofen [4]. In that study, a single 60 mg dose of loxoprofen led to a mean temperature reduction of 1.25 °C, with a standard deviation of 0.69 °C at the 4 h mark. According to the following parameters, a non-inferiority margin of −0.35 °C, a one-sided significance level of 0.025, and a power of 90%, the required sample size was 82 participants per group. Accounting for a 10% dropout rate, the final target sample size was set at 92 participants per group.

Statistical analysis was conducted using both the per-protocol set (PPS) and the full analysis set (FAS). The FAS consisted of participants who had been administered the study drug at least once and had data available for the primary efficacy outcome at least once after administration. In the FAS, if a missing efficacy outcome occurred during the trial or participants dropped out before the end of the trial, the missing value was imputed using the last observation carried forward (LOCF) method. However, baseline values were not used for replacement. The PPS included the participants who met all the inclusion criteria and completed the study according to the protocol. The data on efficacy were analyzed using the PPS, with additional analysis performed with the FAS. Demographic and safety analyses were based on a safety set that included participants who received at least one dose of the study medication. Missing values for the PPS and safety analysis sets were excluded. Non-inferiority of pelubiprofen to Loxoprofen was established if the lower bound of the one-sided 97.5% confidence interval (CI) of the mean difference in axillary temperature reduction at 4 h post-dose exceeded −0.35.

Statistical differences in demographics, safety profiles, and secondary efficacy outcomes between the two groups were analyzed using *t*-tests for continuous variables and chi-square or Fisher’s exact tests for categorical variables. Within-group differences in continuous variables were analyzed using paired *t*-tests.

## 3. Results

### 3.1. Participants

A total of 188 participants were screened, and 183 were randomized to receive either pelubiprofen (*n* = 91) or loxoprofen (*n* = 92) (Figure 1). Five participants were excluded during screening as they did not meet the inclusion criteria or withdrew their consent. Of the one hundred eighty-three participants, two in the pelubiprofen group withdrew from the study without medication. Therefore, the safety set included 181 participants, with 89 in the pelubiprofen group and 92 in the loxoprofen group, forming the FAS. A total of 174 participants completed the study; however, based on the PPS criteria, the PPS included 61 participants in the pelubiprofen group and 69 participants in the loxoprofen group.

### 3.2. Baseline Characteristics

There were no significant differences in the demographic or clinical characteristics between the groups (*p*-value > 0.05) (Table 1). The mean ± SD for age was 38.10 ± 16.44 years in the pelubiprofen group and 37.57 ± 16.22 years in the loxoprofen group. Females accounted for 59.55% of the pelubiprofen group and 54.35% of the loxoprofen group. The mean ± SD for the duration of illness at enrollment was 1.04 ± 0.72 days in the pelubiprofen group and 1.14 ± 0.69 days in the loxoprofen group.

### 3.3. Primary Outcome

**Figure 2:**
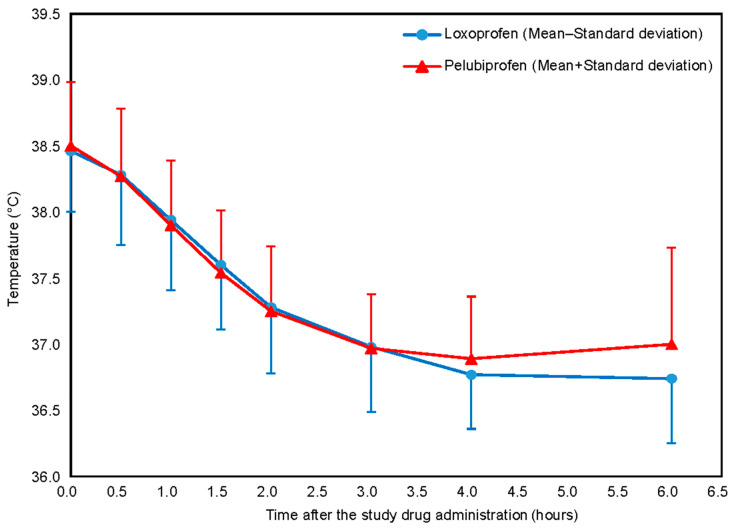
Mean axillary temperature changes from baseline to 6 h post-dose.

At baseline, the mean axillary temperatures were 38.50 ± 0.48 °C in the pelubiprofen group and 38.46 ± 0.46 °C in the loxoprofen group (Table 2b, Figure 2). At 4 h post-dose, temperatures decreased to 36.89 ± 0.47 °C and 36.77 ± 0.41 °C, respectively. The mean reduction from baseline was −1.61 ± 0.62 °C for pelubiprofen and −1.69 ± 0.62 °C for loxoprofen. The between-group difference was 0.08 °C (one-sided 97.5% CI, −0.30 °C to ∞) (Table 2a), satisfying the non-inferiority criterion. Within each group, the decrease in temperature from baseline was statistically significant (*p* < 0.0001) (Table 2b). The statistical significance observed in the FAS analysis was consistent with that in the PPS analysis (Table S1-1, S1-2).

### 3.4. Secondary Outcomes

In the assessment of secondary outcomes related to temperature, the mean ± SD for the AUC for axillary temperature over 6 h was −7.28 ± 2.83 in the pelubiprofen group and −7.41 ± 2.96 in the loxoprofen group, with no significant differences between the groups (*p* = 0.8004). Similarly, the maximum temperature reduction at 6 h did not differ significantly (−1.79 ± 0.59 vs. −1.84 ± 0.59, *p* = 0.6502; Table 2c). The proportion of participants achieving temperature normalization, defined as a temperature below 37 °C within 6 h, also showed no significant difference between the groups (80.33% vs. 81.16%, *p* = 0.9044; Table 2, Table 3 and Table 4). Additionally, the time to temperature normalization was comparable across both groups (3.29 ± 1.41 vs. 3.19 ± 1.08; *p* = 0.6858) (Table 2, Table 3 and Table 4).

**Figure 3:**
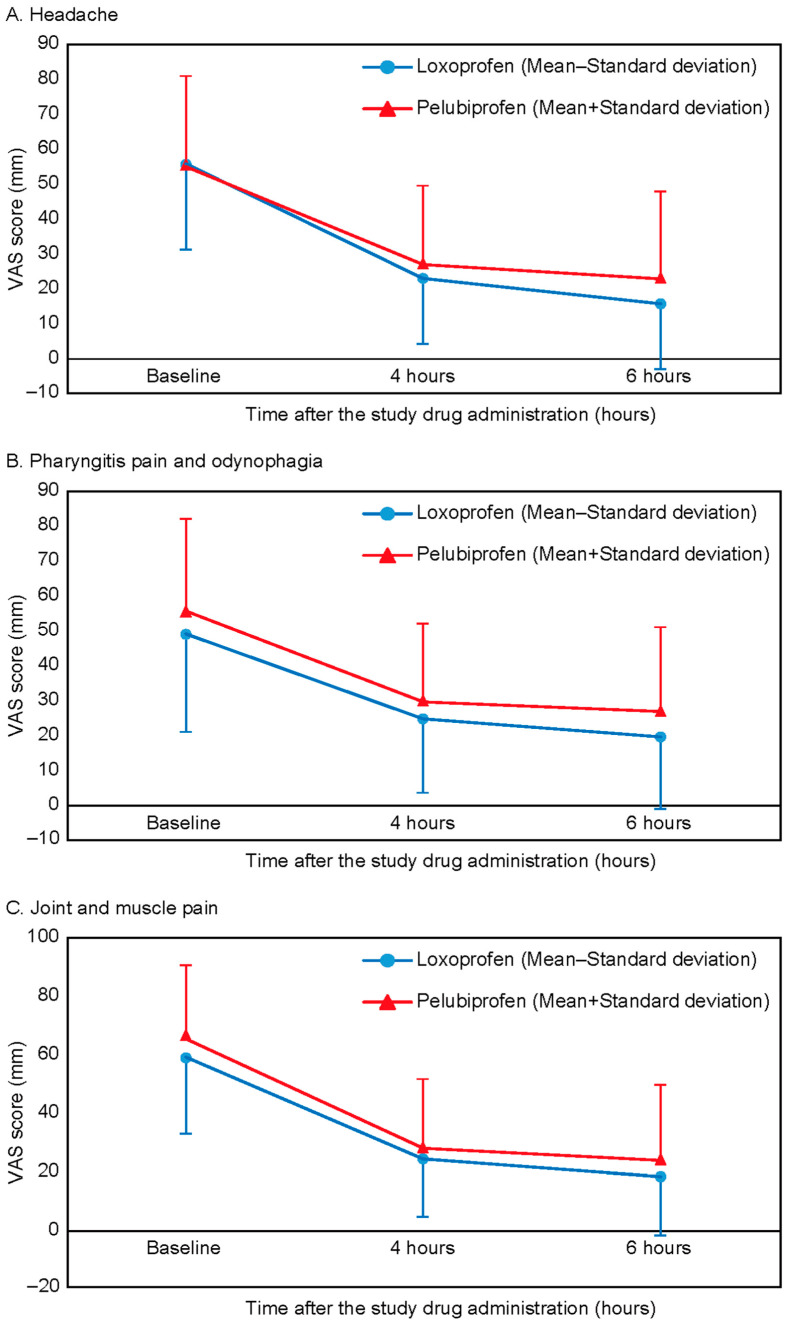
Mean VAS pain score changes from baseline to 6 h post-dose.

For pain-related outcomes, headache severity showed significant reductions within each group at 4 and 6 h post-dose compared to baseline (*p* < 0.0001). However, no significant differences were observed between the groups (−28.16 ± 21.44 vs. −32.64 ± 20.29; *p* = 0.2241 at 4 h, −32.51 ± 29.66 vs. −39.93 ± 23.72; *p* = 0.1159 at 6 h; Table 3a, Figure 3A). Similar trends were observed for reductions in pharyngeal pain/odynophagia and joint/muscle pain, with significant improvements within each group but no significant differences between the groups at either time point (Table 3b,c, Figure 3B,C). The statistical significance from the FAS was similar to the results from the PPS (Tables S1-3, S1-4, S2-1, S2-2, and S2-3).

### 3.5. Safety Assessment

Safety assessments were conducted for all participants who received at least one dose of the study medication (*n* = 181). In total, nine AEs occurred in seven participants (three AEs in three participants in the pelubiprofen group and six AEs in four participants in the loxoprofen group; Table 4). The majority of AEs were mild in severity, and seven of the nine AEs were reported. In the pelubiprofen group, two AEs were mild, and one was moderate (‘pyrexia’). In the loxoprofen group, five AEs were mild, and one was severe (‘hepatitis A’).

One adverse drug reaction (ADR) occurred in the pelubiprofen group (1.12%); it was dyspepsia, an expected ADR based on the MFDS label for pelubiprofen, where dyspepsia is commonly reported (≥1/100 to <1/10). No ADRs were reported in the loxoprofen group. Serious adverse events (SAEs) were observed in one participant in the pelubiprofen group (1.12%) and four participants in the loxoprofen group (4.35%), totaling one SAE in the pelubiprofen group and five SAEs in the loxoprofen group. The SAE in the pelubiprofen group was pyrexia (moderate severity), and those in the loxoprofen group included hepatitis A (severe severity), influenza, liver abscess, pneumonia, and papillary thyroid cancer. None of the SAEs was related to the study medications. Except for pyrexia in the pelubiprofen group and hepatitis A in the loxoprofen group, all the SAEs were mild. All SAEs, except papillary thyroid cancer, in the loxoprofen group were resolved without sequelae.

Laboratory assessments and physical examinations showed no clinically significant differences between the groups. Minor differences in monocyte, eosinophil, and basophil counts were observed but were not clinically significant. Vital signs remained stable throughout the study.

## 4. Discussion

This randomized trial demonstrated that pelubiprofen is non-inferior to loxoprofen in reducing fever associated with URTIs. The secondary efficacy outcomes, including pain relief and other temperature-related measures, were comparable between the two groups. Additionally, the safety profiles of pelubiprofen and loxoprofen were similar, with no significant safety concerns observed.

The primary finding of our study was the non-inferiority of pelubiprofen to loxoprofen in reducing fever associated with URTIs. The mean reduction in axillary temperature at 4 h post-dose met and closely aligned with the predefined non-inferiority margin, demonstrating that pelubiprofen effectively reduced fever in patients with URTIs.

Our findings align with those of previous studies evaluating the antipyretic effects of NSAIDs in patients with URTIs. A study investigating aspirin and acetaminophen in febrile adults reported mean maximum temperature reductions of 1.32 °C and 1.67 °C for aspirin (500 and 1000 mg) and 1.25 °C and 1.71 °C for acetaminophen (500 and 1000 mg). This reduction is similar to the −1.61 ± 0.62 °C observed with pelubiprofen in our study [5]. These findings support the antipyretic efficacy of pelubiprofen, which is comparable to that of other commonly used NSAIDs for treating URTIs.

The route of administration can influence both the onset and magnitude of antipyretic effects. For instance, intravenous propacetamol achieved a more rapid reduction in body temperature than oral dexibuprofen within the first 2 h of administration [6], likely due to differences in absorption rates and the time required to reach peak plasma concentration. Although pelubiprofen is administered orally, its rapid onset of action, with significant temperature reductions observed as early as 1 h of dosing, highlights its effectiveness in acute settings.

The consistent antipyretic effects observed across various studies reinforce the validity of our findings. The efficacy of pelubiprofen is comparable to that of other NSAIDs and acetaminophen, providing clinicians with an effective alternative for managing patients with URTIs.

Secondary efficacy outcomes, including temperature changes over 6 h and pain relief, were similar between the two groups. The reductions in VAS scores for headache, pharyngeal pain/odynophagia, and joint/muscle pain were clinically significant, with decreases exceeding 10 mm, which is considered a minimal clinically important difference [7]. Our findings align with those of previous studies demonstrating similar pain relief with other NSAIDs in febrile patients. An earlier study reported that dexibuprofen provided effective pain relief in febrile patients, with VAS score reductions similar to our observations with pelubiprofen [6]. The comparable analgesic efficacy of pelubiprofen indicates its effectiveness in alleviating multiple symptoms associated with URTI symptoms and enhancing patient comfort. Its dual antipyretic and analgesic properties enable comprehensive symptom management.

The safety profiles of pelubiprofen and loxoprofen are comparable, with a low incidence of AEs and no unexpected safety concerns. Most AEs were mild and resolved without sequelae. Laboratory tests, including assessments of monocytes, eosinophils, and basophils, revealed statistically significant differences, but these differences were not clinically meaningful, as all values remained within normal ranges. Consistent with prior research, our safety findings indicate that NSAIDs are generally well tolerated when used appropriately. The PRECISION trial demonstrated that some NSAIDs did not have higher cardiovascular risks than others, highlighting the need for individualized assessment of the safety profile of each NSAID. Although we did not investigate long-term cardiovascular or gastrointestinal risks, the absence of SAEs supports the short-term use of pelubiprofen in the treatment of URTIs.

The importance of managing fever in clinical settings, particularly during conditions like coronavirus disease 2019 (COVID-19) and in emergency care, cannot be overstated [8]. Effective fever reduction alleviates patient discomfort and also influences the perceptions of clinicians regarding illness severity and prognosis, potentially leading to more effective and timely treatment strategies. A notable aspect of our study is the exclusion of patients diagnosed with influenza. Influenza can present with more severe symptoms and may respond differently to antipyretic therapy compared to other URTIs. By excluding these patients, we aimed to reduce variability and focus on the typical URTI population.

Despite the promising findings, this study has some limitations. First, this study was conducted exclusively in South Korea under a single-dose protocol and limited observation period, potentially restricting the generalizability of our findings to other populations and limiting our capacity to thoroughly evaluate pelubiprofen’s long-term efficacy and safety. Future research with extended follow-up and repeated dosing in broader, more diverse genetic backgrounds or febrile conditions beyond URTIs is warranted to elucidate pelubiprofen’s safety profile and the durability of its antipyretic and analgesic effects.

Additionally, although we demonstrated the non-inferiority of pelubiprofen for fever reduction compared with loxoprofen, our focus primarily on fever and certain pain indicators precludes broader conclusions about other clinically relevant URTI symptoms or cost-effectiveness. Further investigations involving comprehensive symptom assessments, economic evaluations, and larger, more diverse patient cohorts could help define pelubiprofen’s full clinical utility.

Lastly, our sample size was designed to establish non-inferiority in fever reduction and may still have been relatively limited for detecting rare or subtle adverse events, thus lacking the statistical power to detect rare or subtle adverse events. Increasing the sample size in subsequent trials will enhance statistical power for identifying infrequent safety signals and provide strong evidence for the clinical use of pelubiprofen.

## 5. Conclusions

Pelubiprofen is not inferior to loxoprofen in reducing fever in patients with acute URTIs and provides comparable pain relief with a similar safety profile. These findings suggest that pelubiprofen is an effective and safe NSAID for managing URTI symptoms. Further studies evaluating the long-term use of pelubiprofen and comparing it with other antipyretic agents would be beneficial to fully establish its clinical use.

## Figures and Tables

**Table 1:** Demographic and clinical characteristics at baseline *.

	Pelubiprofen Group (N = 89)	Loxoprofen Group (N = 92)	*p*-Value
**Sex**			
Male	36 (40.45)	42 (45.65)	0.4798 ^(1)^
Female	53 (59.55)	50 (54.35)	
**Age (years)**	38.10 ± 16.44	37.57 ± 16.22	0.8255 ^(2)^
**Age group**			
15~29	33 (37.08)	35 (38.04)	0.9337 ^(1)^
30~39	23 (25.84)	27 (29.35)	
40~49	14 (15.73)	11 (11.96)	
50~59	4 (4.49)	5 (5.43)	
≥60	15 (16.85)	14 (15.22)	
**Duration of illness (days)**	1.04 ± 0.72	1.14 ± 0.69	0.3592 ^(2)^
**Previous history (Yes)**	36 (40.45)	34 (36.96)	0.6295 ^(1)^
**Current medical history (Yes)**	32 (35.96)	27 (29.35)	0.3431 ^(1)^
**Surgical history (Yes)**	4 (4.49)	2 (2.17)	0.4388 ^(3)^

* Data from participants who were administered the study drug. Duration of illness = Visit date − date of diagnosis of Acute upper respiratory tract infection diagnosis date. Age, Duration of illness: Mean ± Standard deviation. Sex, Age group, Previous history, Current medical history, Surgical history: Frequency (Percentage). ^(1)^ Chi-square test, ^(2)^ *t*-test, ^(3)^ Fisher’s exact test.

**Table 2:** (**a**) Temperature reduction at 4 h post-dose. (**b**) Temperature at baseline and after study drug administration. (**c**) AUC and maximum temperature reduction. (**d**) Fever resolution post-study drug administration.

(**a**)							
**Comparison**	**Mean ± SD**			**97.5% CI**			
Loxoprofen–Pelubiprofen	−0.0800 ± 0.62			(−0.30, ∞]			
(**b**)							
**Time**	**Pelubiprofen (N = 61)**			**Loxoprofen (N = 69)**			
	**Mean ± SD**	**Median**	**Min~Max**	**Mean ± SD**	**Median**	**Min~Max**	***p*-Value ^(1)^**
Baseline	38.50 ± 0.48	38.40	38.00~39.70	38.46 ± 0.46	38.30	38.00~39.70	0.6462
0.5 h post-dose	38.27 ± 0.51	38.10	37.60~39.80	38.28 ± 0.53	38.10	37.50~39.80	0.8660
1 h post-dose	37.90 ± 0.49	37.80	36.90~39.40	37.94 ± 0.53	37.90	37.00~39.90	0.6279
1.5 h post-dose	37.54 ± 0.47	37.50	36.60~38.70	37.60 ± 0.49	37.60	36.70~39.80	0.4568
2 h post-dose	37.25 ± 0.49	37.20	36.40~38.50	37.28 ± 0.50	37.30	36.40~38.80	0.7004
3 h post-dose	36.97 ± 0.41	37.00	36.20~38.00	36.98 ± 0.49	37.00	36.10~38.60	0.8791
4 h post-dose	36.89 ± 0.47	36.90	36.00~38.50	36.77 ± 0.41	36.80	36.00~38.50	0.1292
6 h post-dose	37.00 ± 0.73	36.80	36.10~39.80	36.74 ± 0.49	36.60	36.00~38.60	0.0167
Difference (4 h-Baseline)	−1.61 ± 0.62	−1.50	−3.60~−0.30	−1.69 ± 0.62	−1.70	−3.40~0.10	0.4671
*p*-value ^(2)^	<0.0001			<0.0001			
(**c**)							
	**Pelubiprofen (** **N = 61)**			**Loxoprofen (** **N = 69)**			
	**Mean ± SD**	**Median**	**Min~Max**	**Mean ± SD**	**Median**	**Min~Max**	***p*-Value ^(1)^**
AUC (0–6 h)	−7.28 ± 2.83	−6.90	−16.15~2.15	−7.41 ± 2.96	−7.25	−15.98~0.73	0.8004
Maximum temperature reduction	−1.79 ± 0.59	−1.70	−3.60~−0.80	−1.84 ± 0.59	−1.80	−3.50~−0.30	0.6502
(**d**)							
**Time**			**Pelubiprofen (** **N = 61)**		**Loxoprofen (** **N = 69)**		***p*-Value**
Normalization rate (over 6 h)			49 (80.33%)		56 (81.16%)		0.9044 ^(3)^
Normalization rate (0.5 h)			0 (0.00%)		0 (0.00%)		NA
Normalization rate (1 h)			1 (1.64%)		0 (0.00%)		0.4692 ^(4)^
Normalization rate (1.5 h)			4 (6.56%)		3 (4.35%)		0.7055 ^(4)^
Normalization rate (2 h)			15 (24.59%)		15 (21.74%)		0.7002 ^(3)^
Normalization rate (3 h)			28 (45.90%)		34 (49.28%)		0.7007 ^(3)^
Normalization rate (4 h)			37 (60.66%)		52 (75.36%)		0.0717 ^(3)^
Normalization rate (6 h)			39 (63.93%)		48 (69.57%)		0.4959 ^(3)^
Time to normalization			3.29 ± 1.41		3.19 ± 1.08		0.6858 ^(1)^

AUC: Area under the curve. SD: Standard deviation. CI: Confidence interval for the mean difference between the two groups. Number (percentage); NA: Not applicable. Mean ± Standard deviation. ^(1)^ *t*-test. ^(2)^ Paired *t*-test. ^(3)^ Chi-square test. ^(4)^ Fisher’s exact test.

**Table 3:** (**a**) Headache at baseline and after study drug administration. (**b**) Pharyngeal pain and odynophagia at baseline and after drug administration. (**c**) Joint and muscle pain.

(**a**)							
**Time**	**Pelubiprofen (N = 61)**			**Loxoprofen (N = 69)**			
	**Mean ± SD**	**Median**	**Min~Max**	**Mean ± SD**	**Median**	**Min~Max**	***p*-Value ^(1)^**
Baseline	55.30 ± 25.55	58.00	0.00~100.00	55.55 ± 24.22	60.00	0.00~100.00	0.9534
4 h post-dose	27.13 ± 22.59	20.00	0.00~90.00	22.91 ± 18.69	20.00	0.00~80.00	0.2464
6 h post-dose	22.79 ± 25.06	15.00	0.00~100.00	15.62 ± 18.24	10.00	0.00~80.00	0.0682
Difference (4 h-Baseline)	−28.16 ± 21.44	−27.00	−85.00~15.00	−32.64 ± 20.29	−30.00	−80.00~1.00	0.2241
Difference (6 h-Baseline)	−32.51 ± 29.66	−30.00	−90.00~60.00	−39.93 ± 23.72	−40.00	−80.00~10.00	0.1159
*p*-value (4 h-Baseline) ^(2)^	<0.0001			<0.0001			
*p*-value (6 h-Baseline) ^(2)^	<0.0001			<0.0001			
(**b**)							
**Time**	**Pelubiprofen (N = 61)**			**Loxoprofen (N = 69)**			***p*-Value ^(1)^**
	**Mean ± SD**	**Median**	**Min~Max**	**Mean ± SD**	**Median**	**Min~Max**	
Baseline	55.80 ± 26.46	60.00	0.00~100.00	49.13 ± 28.24	50.00	0.00~100.00	0.1685
4 h post-dose	29.79 ± 22.40	28.00	0.00~90.00	24.84 ± 21.11	20.00	0.00~80.00	0.1974
6 h post-dose	26.90 ± 24.49	23.00	0.00~90.00	19.59 ± 20.69	10.00	0.00~80.00	0.0676
Difference (4 h-Baseline)	−26.02 ± 24.61	−21.00	−90.00~50.00	−24.29 ± 21.17	−20.00	−91.00~1.00	0.6679
Difference (6 h-Baseline)	−28.90 ± 29.58	−30.00	−90.00~50.00	−29.54 ± 24.08	−30.00	−100.00~21.00	0.8930
*p*-value (4 h-Baseline) ^(2)^	<0.0001			<0.0001			
*p*-value (4 h-Baseline) ^(2)^	<0.0001			<0.0001			
(**c**)							
**Time**	**Pelubiprofen (N = 61)**			**Loxoprofen (N = 69)**			***p*-Value ^(1)^**
	**Mean ± SD**	**Median**	**Min~Max**	**Mean ± SD**	**Median**	**Min~Max**	
Baseline	65.98 ± 24.91	70.00	0.00~100.00	59.03 ± 25.82	60.00	0.00~100.00	0.1217
4 h post-dose	27.67 ± 24.13	20.00	0.00~80.00	24.20 ± 19.50	25.00	0.00~70.00	0.3667
6 h post-dose	23.69 ± 26.16	10.00	0.00~90.00	17.86 ± 19.80	10.00	0.00~84.00	0.1587
Difference (4 h-Baseline)	−38.31 ± 22.16	−40.00	−100.00~1.00	−34.83 ± 22.32	−30.00	−90.00~0.00	0.3742
Difference (6 h-Baseline)	−42.30 ± 25.72	−42.00	−100.00~10.00	−41.17 ± 24.32	−40.00	−100.00~0.00	0.7989
*p*-value (4 h-Baseline) ^(2)^	<0.0001			<0.0001			
*p*-value (6 h-Baseline) ^(2)^	<0.0001			<0.0001			

SD: Standard deviation. ^(1)^ *t*-test. ^(2)^ Paired *t*-test.

**Table 4:** Adverse events.

SOC	Pelubiprofen (N = 89)	Loxoprofen (N = 92)
Infections and infestations	0 (0)	3 (4)
General disorders and administration site conditions	1 (1)	1 (1)
Blood and lymphatic system disorders	1 (1)	0 (0)
Gastrointestinal disorders	1 (1)	0 (0)
Neoplasms benign, malignant, and unspecified	0 (0)	1 (1)
Total	3 (3)	4 (6)

Frequency of adverse events (serious adverse events). SOC: System Organ Class.

## Data Availability

The dataset supporting the conclusions of this article is included in the article.

## Supplementary Materials

The following supporting information can be downloaded at https://www.mdpi.com/article/10.3390/jcm14051450/s1. Table S1: Table S1-1. Temperature reduction at 4 h post-dose; Table S1-2. Temperature at baseline and after study drug administration; Table S1-3. AUC and maximum temperature reduction; Table S1-4. Fever resolution post-study drug administration; Table S2-1. Headache at baseline and after study drug administration; Table S2-2. Pharyngeal pain and odynophagia at baseline and after drug administration; Table S2-3. Joint and muscle pain.
