# Supplementary material for: The Efficacy and Safety of Pelubiprofen in the Treatment of Acute Upper Respiratory Tract Infection: A Multicenter, Randomized, Double-Blind, Non-Inferiority Phase III Clinical Trial Compared to Loxoprofen
Source: J Clin Med. 2025 Feb 21;14(5):1450. doi: 10.3390/jcm14051450 (PMC11901028; doi:10.3390/jcm14051450)
Supplement: Supplementary file 1 [file jcm-14-01450-s001.zip › jcm-3438856-supplementary.pdf]

Supplementary Materials

Table S1-1. Temperature reduction at 4 hours post-dose

Comparison	mean ± SD	97.5% CI
Loxoprofen - Pelubiprofen	-0.0500± 0.69	(-0.25, ∞]

SD: Standard deviation
CI: Confidence interval for the mean difference between the two groups

Table S1-2. Temperature at baseline and after study drug administration

Time	Pelubiprofen (N=89)			Loxoprofen (N=92)			p-value[1)]
	mean ± SD	median	min ~ max	mean ± SD	median	min ~ max	
Baseline	38.52 ± 0.52	38.4	38.00 ~ 39.90	38.49 ± 0.51	38.3	38.00 ~ 39.70	0.7592
0.5 hours post-dose	38.31 ± 0.57	38.1	37.60 ~ 40.00	38.29 ± 0.54	38.1	37.50 ~ 39.80	0.7680
1-hour post-dose	37.93 ± 0.55	37.8	36.90 ~ 39.80	37.99 ± 0.55	37.9	37.00 ~ 39.90	0.4825
1.5 hours post-dose	37.56 ± 0.50	37.5	36.60 ~ 39.00	37.65 ± 0.51	37.6	36.70 ~ 39.80	0.2490
2 hours post-dose	37.27 ± 0.52	37.2	36.20 ~ 38.50	37.33 ± 0.51	37.3	36.40 ~ 38.80	0.4325
3 hours post-dose	36.98 ± 0.43	37.0	36.20 ~ 38.00	37.02 ± 0.47	37.0	36.10 ~ 38.60	0.5285
4 hours post-dose	36.90 ± 0.56	36.8	36.00 ~ 39.10	36.83 ± 0.46	36.8	36.00 ~ 38.50	0.3367
6 hours post-dose	37.04 ± 0.83	36.8	36.00 ~ 39.80	36.75 ± 0.50	36.6	36.00 ~ 38.60	0.0052
Difference (4 hours - Baseline)	-1.61 ± 0.72	-1.6	-3.60 ~ 0.90	-1.66 ± 0.67	-1.6	-3.50 ~ 0.10	0.6319
p-value[2)]	<0.0001			<0.0001			

SD: Standard deviation
[1)] t-test
[2)]paired t-test

Table S1-3. AUC and maximum temperature reduction

	Pelubiprofen (N=89)			Loxoprofen (N=92)			p-value[1)]
	**mean ± SD**	**median**	**min ~ max**	**mean ± SD**	**median**	**min ~ max**	
AUC (0-6 hours)	-7.22 ± 3.14	-7.43	-16.15 ~ 1.95	-7.16 ± 3.24	-7.10	-15.98 ~ 0.73	0.9004
Maximum temperature reduction	-1.82 ± 0.64	-1.70	-3.60 ~ -0.30	-1.81 ± 0.64	-1.80	-3.50 ~ -0.30	0.9229

AUC: Area under the curve. SD: Standard deviation
[1)] t-test

Table S1-4. Fever resolution post-study drug administration

Time	Pelubiprofen (N=89)	Loxoprofen (N=92)	p-value
Normalization rate (over 6 hours)	67 (75.28%)	69 (75.00%)	0.9651[1)]
Normalization rate (0.5 hours)	0 (0.00%)	0 (0.00%)	NA
Normalization rate (1 hour)	1 (1.12%)	0 (0.00%)	0.4917[2)]
Normalization rate (1.5 hours)	5 (5.62%)	3 (3.26%)	0.4920[2)]
Normalization rate (2 hours)	23 (25.84%)	18 (19.57%)	0.3131[1)]
Normalization rate (3 hours)	42 (47.19%)	39 (42.39%)	0.5162[1)]
Normalization rate (4 hours)	55 (61.80%)	62 (67.39%)	0.4313[1)]
Normalization rate (6 hours)	54 (61.80%)	60 (65.22%)	0.5268[1)]
Time to normalization	3.14 ± 1.30	3.21 ± 1.03	0.7350[3)]

Number (percentage); NA: Not Applicable
Mean ± Standard deviation
[1)]Chi-square test
[2)]Fisher's exact test
[3)]t-test

Table S2-1. Headache at baseline and after study drug administration

Time	Pelubiprofen (N=89)			Loxoprofen (N=92)			p-value[1)]
	**mean ± SD**	**median**	**min ~ max**	**mean ± SD**	**median**	**min ~ max**	
Baseline	56.54 ± 25.30	65	0.00 ~ 100.00	54.48 ± 25.42	60	0.00 ~ 100.00	0.5853
4 hours post-dose	25.96 ± 21.32	20	0.00 ~ 90.00	22.46 ± 18.84	20	0.00 ~ 80.00	0.2481
6 hours post-dose	21.87 ± 24.28	15	0.00 ~ 100.00	15.62 ± 18.65	10	0.00 ~ 80.00	0.0560
Difference (4 hours - Baseline)	-30.58 ± 22.86	-29	-89.00 ~ 15.00	-33.71 ± 22.21	-30	-90.00 ~ 1.00	0.7399
Difference (6 hours - Baseline)	-34.67 ± 29.38	-30	-91.00 ~ 60.00	-38.55 ± 24.52	-40	-90.00 ~ 10.00	0.3406
p-value (4 hours - Baseline)[2)]	<0.0001			<0.0001			
p-value (6 hours – Baseline)[2)]	<0.0001			<0.0001			

SD: Standard deviation

[1)] t-test

[2)]paired t-test

Table S2-2. Pharyngeal pain and odynophagia at baseline and after drug administration

Time	Pelubiprofen (N=89)			Loxoprofen (N=92)			p-value[1)]
	**mean ± SD**	**median**	**min ~ max**	**mean ± SD**	**median**	**min ~ max**	
Baseline	51.72 ± 26.15	54	0.00 ~ 100.00	50.39 ± 28.31	50	0.00 ~ 100.00	0.7437
4 hours post-dose	27.80 ± 21.91	20	0.00 ~ 90.00	25.39 ± 21.25	20	0.00 ~ 80.00	0.4584
6 hours post-dose	24.63 ± 22.81	20	0.00 ~ 90.00	20.74 ± 22.14	10	0.00 ~ 90.00	0.2501
Difference (4 hours - Baseline)	-23.92 ± 22.28	-22	-90.00 ~ 50.00	-24.65 ± 21.29	-20	-91.00 ~ 1.00	0.8234

Time	Pelubiprofen (N=89)			Loxoprofen (N=92)			p-value[1)]
	mean ± SD	median	min ~ max	mean ± SD	median	min ~ max	
Difference (6 hours - Baseline)	-27.09 ± 27.50	-24	-90.00 ~ 50.00	-29.30 ± 23.98	-30	-100.00 ~ 21.00	0.5678
p-value (4 hours – Baseline)[2)]	<0.0001			<0.0001			
p-value (4 hours – Baseline)[2)]	<0.0001			<0.0001			

SD: Standard deviation
[1)] t-test
[2)]paired t-test

Table S2-3. Joint and muscle pain

Time	Pelubiprofen (N=89)			Loxoprofen (N=92)			p-value[1)]
	mean ± SD	median	min ~ max	mean ± SD	median	min ~ max	
Baseline	64.53 ± 25.90	70	0.00 ~ 100.00	60.79 ± 26.00	61	0.00 ~ 100.00	0.3344
4 hours post-dose	26.42 ± 22.16	20	0.00 ~ 80.00	25.66 ± 19.82	25	0.00 ~ 70.00	0.8115
6 hours post-dose	21.30± 23.83	10	0.00 ~ 90.00	18.35 ± 19.76	10	0.00 ~ 84.00	0.3690
Difference (4 hours - Baseline)	-38.11 ± 23.24	-40	-100.00 ~ 1.00	-35.26 ± 23.50	-30	-100.00 ~ 0.00	0.4163
Difference (6 hours - Baseline)	-43.22 ± 27.05	-47	-100.00 ~ 10.00	-42.57 ± 25.84	-40	-100.00 ~ 10.00	0.8697
p-value (4 hours – Baseline)[2)]	<0.0001			<0.0001			
p-value (6 hours – Baseline)[2)]	<0.0001			<0.0001			

SD: Standard deviation
[1)] t-test
[2)]paired t-test
